# Knee osteotomies significantly influence coronal ankle alignment: A radiographic analysis

**DOI:** 10.1002/jeo2.70252

**Published:** 2025-05-05

**Authors:** Alessio Maione, Carlo Minoli, Matteo Davide Parmigiani, Martino Travi, Filippo Calanna, Daniele Marcolli, Riccardo Compagnoni, Paolo Ferrua, Massimo Berruto, Pietro Simone Randelli

**Affiliations:** ^1^ U.O.C. 1st Orthopedic Clinic, ASST Centro Specialistico Ortopedico Traumatologico Gaetano Pini‐CTO Milan Italy; ^2^ U.O.C. Week Surgery, ASST Centro Specialistico Ortopedico Traumatologico Gaetano Pini‐CTO Milan Italy; ^3^ Residency Program in Othopedics and Traumatology University of Milan Milan Italy; ^4^ Department of Biomedical, Surgical and Dental Sciences University of Milan Milan Italy; ^5^ Department of Biomedical Sciences for Health University of Milan Milan Italy

**Keywords:** coronal ankle alignment, knee, knee osteotomy, lower limb alignment, radiographs

## Abstract

**Purpose:**

This study aimed to evaluate the effect of lateral closing‐wedge high tibial osteotomy (LCW‐HTO) and medial closing‐wedge distal femoral osteotomy (MCW‐DFO) on tibio‐talar inclination (TTI) and Mikulicz lateral distal tibial angle (M‐LDTA). We hypothesized that knee osteotomies significantly alter ankle coronal alignment by modifying TTI and distal tibial alignment in relation to the mechanical axis.

**Methods:**

A retrospective radiographic analysis was conducted on 60 knees from 52 patients (37 LCW‐HTO and 23 MCW‐DFO) treated between 2006 and 2020. Inclusion criteria included full‐length weight‐bearing radiographs pre‐ and post‐operatively, no prior ipsilateral lower limb surgery, absence of shaft deformities or advanced ankle osteoarthritis (Takakura grade >1), and age ≥16 years with ≥2 years of follow‐up. Radiographic parameters measured included LDTA, hip‐knee‐ankle angle, M‐LDTA and TTI, with ankle realignment quantified through differences between LDTA and M‐LDTA and between pre‐ and post‐operative TTI.

**Results:**

In the MCW‐DFO group, the difference between LDTA and M‐LDTA decreased from 3.5 ± 2.3° to 1.3 ± 1.1° (*p* < 0.0001), indicating improved alignment. The LCW‐HTO group showed a smaller but significant reduction from 4.5 ± 1.8° to 2.2 ± 1.7° (*p* < 0.0001). TTI improved significantly in both groups, with a greater adjustment in MCW‐DFO (ΔTTI = 7.0 ± 4.3°, *p* < 0.01) compared to LCW‐HTO (ΔTTI = 4.2 ± 2.7°, *p* < 0.01). The difference between LDTA and TTI decreased in both groups, reflecting post‐operative convergence of the mechanical and anatomical axes.

**Conclusion:**

Knee osteotomies significantly influence ankle coronal alignment, particularly modifying TTI and M‐LDTA. Higher‐level osteotomies (MCW‐DFO) exert a greater effect on ankle alignment than LCW‐HTO. Preoperative valgus or varus knee deformities must be carefully evaluated to anticipate post‐operative ankle imbalance. Surgeons should assess full‐length radiographs to prevent unintended malalignment.

**Level of Evidence:**

Level III.

**Abbreviations**
aHKA: arithmetic hip–knee–ankle
CPAK: Coronal Plane Alignment of the Knee
DFO: distal femoral osteotomy
HKA: hip–knee–ankle
HTO: high tibial osteotomy
LCW‐HTO: lateral closing‐wedge high tibial osteotomy
LDTA: lateral distal tibial angle
MCW‐DFO: medial closing‐wedge distal femoral osteotomy
M‐LDTA: Mikulicz lateral distal tibial angle
TKA: total knee arthroplasty
TTI: tibio‐talar inclination

## INTRODUCTION

Osteotomies around the knee are a well‐established surgical procedure for treating axial deformities of the lower limb, especially in the coronal plane [1, 6, 23, 25, 27].

Several studies have assessed the correlation between the correction of the lower limb's load axis and the improvement of knee symptoms [5, 12, 24, 29], focusing all attention exclusively on the treated joint.

Similarly, to perform a supra‐malleolar osteotomy, surgeons typically assess the alignment of the ankle selectively rather than focus on the whole leg axis. The most commonly considered angle, in this field, is the lateral distal tibial angle (LDTA), along with the alignment of the hindfoot and the subtalar joint [18].

However, it has been observed that realignment of the lower limb at the knee often results in changes in the orientation of the tibiotarsal and hindfoot joints [13, 15].

This consideration reflects that malalignment of the lower limb at the knee level leads to a compensatory reorientation of the ankle and hindfoot [8]. It is unclear how much of this variation is distributed to the tibiotarsal joint and how much to the subtalar joint.

The long‐term result of malalignment would be linked to a degeneration of the ankle and subtalar joint [26], leading to a deterioration in the quality of life and functionality [2].

Defining the correlation between the knee and ankle would enable the surgeon to anticipate the effects of knee osteotomies on the tibiotarsal joint, thereby avoiding the creation of more complex collateral deformities.

All the previous considerations have been partially addressed by other authors, particularly about high tibial osteotomy (HTO) for varus and valgus knees [22], even if less is known about distal femoral osteotomy (DFO).

The purpose of this study is to evaluate, with a retrospective radiographic analysis, the effect of lateral closing wedge high tibial osteotomy (LCW‐HTO) and medial closing wedge distal femoral osteotomy (MCW‐DFO) on the coronal alignment of the tibiotarsal joint.

This study hypothesizes that knee osteotomies result in a significant modification in the coronal alignment of the ankle both in LCW‐HTO for varus knees and MCW‐DFO for valgus knees, quantifiable as a change in the tibio‐talar inclination (TTI) and in the distal tibial alignment in relation to the mechanical axis of the lower limb, the so‐called Mikulicz LDTA (M‐LDTA).

## MATERIALS AND METHODS

A retrospective radiological analysis was conducted on a cohort of 52 patients (60 knees) in a single institution. Twenty‐three patients underwent an MCW‐DFO with valgus alignment (hip–knee–ankle [HKA] angle range min 182°, max 194°). Thirty‐seven patients performed an LCW‐HTO with varus alignment (HKA angle range min 166°, max 178°). All the surgeries were performed by two senior knee surgeons between 2006 and 2020 at our department.

The following inclusion criteria were adopted: patient underwent full‐length weight‐bearing radiographs pre‐ and post‐operatively with no previous ipsilateral surgeries on the lower limb, absence of shaft deformities or ankle osteoarthritis (Takakura classification grade >1) and age ≥16 years at the time of surgery and a minimum 2 years of follow‐up.

Patients were excluded from participation in the study in case of previous fractures of the same limb, ipsilateral ankle arthrodesis or ankle replacements.

The study was designed based on the criteria of the Declaration of Helsinki and approved by the local ethical committee (Fondazione IRCCS Ca' Granda Ospedale Maggiore Policlinico – Milano Area 2, Lombardia, Milan – IOGPMB02).

### Radiographical measurements

**Figure 1:**
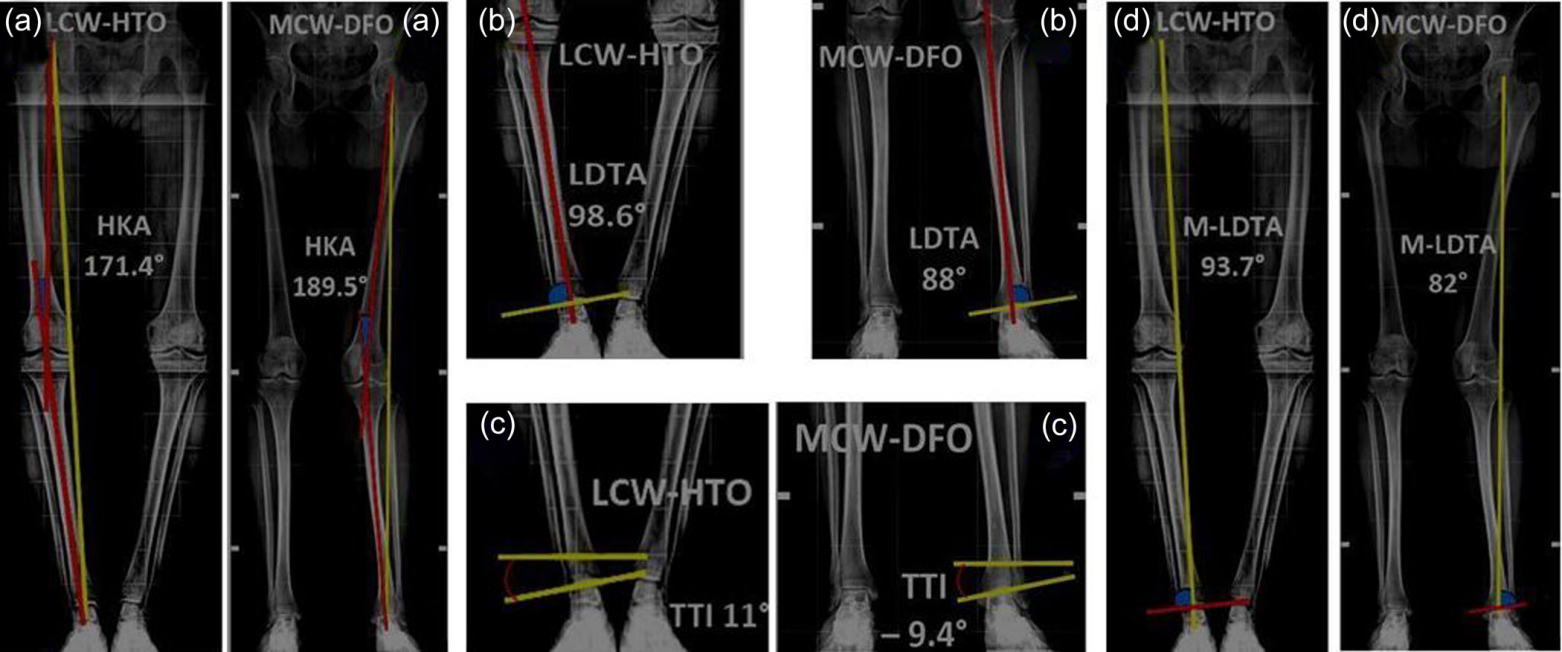
Radiographical measurements. (a) Preoperative hip–knee–ankle (HKA) angle for both groups (in red the mechanical axes, in yellow the Mikulicz line). (b) Preoperative lateral distal tibial angle (LDTA) for both groups. (c) Preoperative tibio‐talar inclination (TTI) for both groups. (d) Preoperative Mikulicz‐LDTA angle (M‐LDTA) for both groups.

Long‐leg anteroposterior weight‐bearing radiographs were collected pre‐ and post‐operatively, using the same PACS system (Agfa Impax 6.0) for every patient (angle measurement resolution 0.1°) (Figure 1). Different angles were measured:

- *HKA angle*: angle between a line from the centre of the femoral head to the centre of the knee joint and the line from the centre of the knee to the centre of the ankle joint (angle measured medially).
- *LDTA*: angle measured between the mid‐diaphyseal line of the tibia and the line parallel to the distal tibial plafond.
- *TTI*: angle between the horizontal line and the tangential line at the talar dome.
- *M‐LDTA*: angle measured between the Mikulicz line and the line parallel to the distal tibial plafond.

The HKA angle, the difference between LDTA and M‐LDTA, the difference between LDTA and TTI and the TTI difference were analyzed in both groups pre‐ and post‐operatively to quantify the realignment of the ankle.

To determine intra‐ and interobserver reliability of radiographic assessments, the measurements were performed twice, at a 2‐month interval, by two orthopaedic surgeons (AM and CM) not involved in the surgical procedure. The intra‐ and interobserver reliability of the radiographic measurements were evaluated using intraclass correlation coefficients (ICCs) using an absolute‐agreement, two‐way mixed‐effects model. According to the ICC values proposed by Koo and Li [16], the reliability was defined as poor (<0.5), moderate (0.5 ≤ ICC < 0.75), good (0.75 ≤ ICC < 0.90), or excellent (≥0.90). The ICC values demonstrated good to excellent reliability, with an intraobserver ICC of 0.92 and an interobserver ICC of 0.89, based on an absolute‐agreement, two‐way mixed‐effects model.

The LDTA is a radiographic parameter that represents the angle formed between the mid‐diaphyseal line of the tibia and the line parallel to the articular surface of the tibial plafond. It is important to emphasize that this angle is not affected by osteotomies, as these procedures do not directly alter the geometry of the distal tibia. However, the LDTA may vary between pre‐ and post‐operative radiographs due to even minimal differences in patient positioning during image acquisition. Changes in limb rotation or radiographic projection can alter the measurement of the LDTA, making it a sensitive indicator of positional variations rather than structural changes.

The specific goal of comparing pre‐ and post‐operative LDTA (calculating the delta) is to quantify the effect of limb rotation or patient positioning on the observed difference in the angle. This helps exclude spurious variations caused by radiographic technique and isolates the true impact of the osteotomy on alignment changes.

To evaluate the true effect of osteotomy on the alignment of the distal segment of the limb, it is essential to consider the difference between the deltas of pre‐ and post‐operative M‐LDTA and those of LDTA. While the M‐LDTA can be influenced by osteotomy, the delta LDTA serves as an internal control to correct for variations caused by patient rotation or positioning.

Comparing the deltas of M‐LDTA and LDTA allows us to:

1. Isolate the effect of osteotomy on the rotation or deviation of the distal segment.
2. Quantify technical errors caused by rotation or positioning in pre‐ and post‐operative radiographs.
3. Ensure greater accuracy in interpreting results related to the alignment of the leg and ankle.

This methodological approach was considered as crucial for precisely understanding how osteotomy influences the biomechanical alignment of the ankle, eliminating confounding factors.

For this reason, *p* values were evaluated only for the HKA angle, the delta between ΔLDTA and ΔM‐LDTA, and the delta between ΔLDTA and ΔTTI. Statistical comparisons were not performed for the other results, as analyzing these data without accounting for the variation introduced by different patient positioning during radiographs would be misleading and result in incorrect conclusions.

### Statistical analysis

Data were reported as mean ± standard deviation (SD). Statistical analysis was conducted using Python 3.7.6 and a two‐tailed Student's *t* test for paired samples, with significance set at *p* < 0.05 and a 95% confidence interval.

The sample size was calculated with a significance level (*α*) of 0.05 and a desired power (1 − *β)* of 80%. Based on previous studies and clinically relevant thresholds for changes in alignment metrics, an effect size of 0.8 was used. According to the formula *N* = (*μ*0 − *μ*1)2*σ*2⋅(*z*1 − *β* + *z*1 −*α*/2)2, the required sample size was determined to be 55 patients.

## RESULTS

In the MCW‐DFO group, the HKA angle significantly decreased, reflecting improved alignment after surgery (*p* < 0.001). Similarly, in the LCW‐HTO group, the HKA angle showed a notable post‐operative improvement (*p* < 0.001).

For the MCW‐DFO group, the difference between the LDTA and M‐LDTA decreased, indicating better alignment. A similar trend was observed in the LCW‐HTO group, although to a lesser extent.

The TTI also changed significantly after surgery, with a greater adjustment observed in the MCW‐DFO group compared to the LCW‐HTO group. Finally, the difference between the LDTA and TTI was reduced in both groups, with statistically significant improvements.

Ankle osteoarthritis was evaluated at the last follow‐up following the Takakura classification. The mean post‐operative value was 1.3 ± 0.5 for the MCW‐DFO group and 1.2 ± 0.4 for the LCW‐HTO group. Based on these results, there was a significant evolution in the degree of ankle osteoarthritis in both the MCW‐DFO group (*p* < 0.05) and the LCW‐HTO group (*p* < 0.01). However, this is a radiological parameter that does not necessarily correlate with greater patient pain. On the contrary, there was no significant difference between inter‐group post‐operative values (*p* = 0.5).

The study population demographics are available in Table 1 and the complete data about the radiological outcomes are reported in Table 2.

**Table 1:** Demographics.

	Overall	MCW‐DFO	LCW‐HTO
No. of patients	52	21	31
No. of knees	60	23	37
Mean follow‐up, years	10.6 ± 3.6	8.3 ± 3.9	12.1 ± 2.4
Age at surgery, years	50.9 ± 9.6	48.1 ± 12.9	52.7 ± 6.4
Gender			
Male	24	6	18
Female	28	15	13

*Note*: Data are expressed as mean ± SD.

Abbreviations: LCW‐HTO, lateral closing wedge high tibial osteotomy; MCW‐DFO, medial closing wedge distal femoral osteotomy; No., number; SD, standard deviation.

**Table 2:** Comparison of the radiological measurements between the two groups, femoral (MCW‐DFO) and tibial (LCW‐HTO), preoperatively and at the last follow‐up.

Parameter	Follow‐up	MCW‐DFO	LCW‐HTO	*p* **value**
		Mean ± SD	Mean ± SD	
HKA	Pre‐op	187.4 ± 3.4 Range (181.5–193.5°)	172.1 ± 3.1 Range (166.5–178.5°)	
	Last FU	180.2 ± 2.6 Range (176–185°)	177.0 ± 3.6 Range (167.5–182.5°)	
	***p* value**	**<0.0001**	**<0.0001**	
LDTA	Pre‐op	85.6 ± 3.9 Range (72.5–90)	90.0 ± 4.5 Range (84.5–100)	
	Last FU	83.9 ± 3.6 Range (75.2–89.8)	88.5 ± 4.9 Range (78.5–97)	
M‐LDTA	Pre‐op	82.2 ± 5.4 Range (66–89)	89.7 ± 4.1 Range (81–100)	
	Last FU	83.9 ± 4.1 Range (72.5–89.6)	88.2 ± 4.8 Range (78.5–100)	
TTI	Pre‐op	−6.8 ± 6.3 Range (−21.7 to 6.7)	5.3 ± 4.1 Range (−2.5 to 13.8)	
	Last FU	−5.5 ± 5.0 Range (−17 to 3.2)	1.6 ± 5.4 Range (−8.5 to 12.8)	
ΔLDTA	Last FU − Pre‐op	3.4 ± 3.1	3.5 ± 2.8	
ΔM‐LDTA	Last FU − Pre‐op	4.7 ± 3.8	2.7 ± 2.6	
ΔTTI	Last FU − Pre‐op	7.0 ± 4.3	4.2 ± 2.7	**<0.01**
Δ (ΔLDTA − ΔM‐LDTA)	Pre‐op	3.5 ± 2.3	4.5 ± 1.8	
	Last FU	1.3 ± 1.1	2.2 ± 1.7	
	***p* value**	**<0.0001**	**<0.0001**	
Δ (ΔLDTA − ΔTTI)	Pre‐op	89.4 ± 3.9	84.8 ± 3.3	
	Last FU	82.2 ± 5.4	86.9 ± 3.3	
	***p* value**	**<0.0001**	**<0.01**	

*Note*: Data are expressed as mean ± SD; statistically significant difference was achieved with a *p* value ≤ 0.05.

Abbreviations: FU, follow‐up; HKA, hip–knee–ankle angle; LDTA, lateral distal tibial angle; M‐LDTA, Mikulicz‐LDTA angle; Pre‐op, preoperative/baseline; TTI, tibio‐talar inclination.

## DISCUSSION

The most important finding of this study is that DFO and HTO influence the coronal alignment of the ankle in terms of TTI and M‐LDTA, highlighting the importance of evaluating the entire lower limb, not just the anatomical section of interest. Typically performed by knee surgeons, knee osteotomies often neglect the ankle's post‐operative condition, while foot and ankle surgeons may not consider joints above the distal tibia. This study aims to bridge the gap between knee and foot/ankle surgeons to better understand lower limb alignment behaviour.

The findings highlight the close interconnection between knee and foot/ankle mechanics, where the alignment and movement of one joint affect the other. By recognizing these interactions, surgeons from both specialities can adopt a more holistic approach to treating lower limb biomechanics. Our study aimed to clarify the true impact of osteotomy on the alignment of the distal limb segment by examining and analyzing the M‐LDTA, avoiding the confounding factors. Bridging the gap between knee and foot/ankle surgeons and deepening our understanding of lower limb alignment behaviour enables more comprehensive, precise and patient‐centred care strategies.

The M‐LDTA is introduced for the first time in this study as a more effective method for evaluating ankle coronal alignment compared to the classical LDTA, especially for deformities above the distal tibia, because it considers the mechanical axis of the lower limb rather than relying solely on the anatomical alignment of the distal tibia. This makes it less influenced by localized variations or positional differences and provides a more comprehensive assessment of the overall alignment and its relationship to the proximal correction. This paper is one of the few studies addressing the impact of both HTO and DFO on ankle alignment, marking a significant contribution to the existing literature.

In the analyzed population, favourable realignment of the lower limb was successfully achieved through either HTO or DFO. In the preoperative femoral group, the HKA angle changed from a mean of 187.4 ± 3.4° to a mean post‐operative angle of 180.2 ± 2.6° (*p* < 0.001). For the tibial group, the HKA angle shifted from a preoperative mean of 172.1 ± 3.1° to a post‐operative mean of 177.0 ± 3.6° (*p* < 0.001).

Ankle alignment was evaluated using the M‐LDTA, TTI and LDTA. The LDTA remained unchanged by either tibial or femoral osteotomies since the distal tibia was not involved in the surgical correction. Differences between pre‐ and post‐operative M‐LDTA and LDTA were significant, showing the impact of osteotomy on the long axis of the lower limb, the Mikulicz line.

It is widely recognized today that the most effective classification systems based on coronal limb alignment have been introduced by Hirschmann et al. [9, 10, 11] and MacDessi et al. [19]. Hirschmann's functional phenotype classification considers the extent of varus or valgus deformity in the femur and tibia separately, emphasizing the disparity between the mechanical alignment of the femur and tibia. On the other hand, the Coronal Plane Alignment of the Knee (CPAK) classification facilitates a personalized approach for preoperative alignment planning, especially useful in total knee arthroplasty (TKA), considering the estimated joint line obliquity and the arithmetic HKA (aHKA). However, neither classification addresses ankle alignment, and despite the potential benefits of developing a unified classification that includes this aspect, they were not utilized.

The results of this study demonstrate that a knee osteotomy either HTO or DFO is responsible for a statistically significant post‐operative convergence of the LDTA and the M‐LDTA (*p* < 0.0001).

The study shows that osteotomies performed higher up on the limb have a greater impact on the ankle, which can be explained by the longer lever arm available for making corrections at the distal end (*p* < 0.01).

Clinically, it is crucial for knee surgeons to assess ankle coronal inclination before correcting knee deformities to avoid potential malalignment of the ankle, which could lead to distal osteoarthritis, gait problems, and instability. Foot and ankle surgeons should evaluate deformities on full‐length weight‐bearing radiographs to avoid missing double‐level deformities or distal deformities influenced by knee alignment. In summary, these data underline once again how long‐leg anteroposterior weight‐bearing radiographs are indispensable in evaluating ankle behaviour as they provide a detailed, functional assessment of lower limb alignment and biomechanics, which is crucial for accurate diagnosis, treatment planning, and monitoring of surgical outcomes.

Previous studies have linked knee osteotomies or TKAs to changes in ankle alignment. Nha et al. [20] in a recent paper reported that medial closing wedge DFO for valgus knee corrected coronal and hindfoot ankle alignments toward neutral. Krause et al. [17] found that HTO influences ankle joint pressure more than DFO and, in general, that the pressure on the ankle is increased in the varus lower limb alignment more than in the valgus one. Kim et al. [14], instead, noted that talar inclination after HTO was influenced by osteoarthritis severity rather than the surgical correction degree. Bernasconi et al. [3], finally, emphasized using the lower limb's mechanical axis to frame ankle characteristics; their study completely agrees with our results on that term.

The ankle–knee relationship has also been studied in TKA, although the impact is still uncertain, and no consensus has been advocated.

Norton et al. [21] observed correlations between knee deformities and hindfoot alignment in TKA patients. Gursu et al. and Shichman et al. evaluated, respectively, 80 and 107 patients with various degrees of varus deformity who underwent TKA, noting that the correction of the knee deformity has in most cases an effect also on the re‐alignment of the ankle joint [7, 28].

Conversely, Desai et al. [4] highlighted that foot deformities can impact knee alignment and recommended considering these deformities during preoperative evaluations for knee deformity planning. The study has limitations, including its retrospective nature and lack of mid‐ to long‐term clinical assessments of the ankle or gait analysis. Future research should focus on the clinical implications of biomechanical corrections. Despite these limitations, this study provides significant insights into the importance of proximal correction of the underlying joint's orientation. It serves as a reminder for knee surgeons to consider the impact of proximal osteotomy on the ankle and for foot surgeons to evaluate the alignment of the entire lower limb using the M‐LDTA, underscoring the interconnected nature of lower limb deformities.

## CONCLUSIONS

Osteotomies around the knee for correction of genu varum or valgus lead to a coronal reorientation of the ankle. This effect is much more evident at higher levels of the osteotomy. A DFO has a greater effect on the ankle coronal orientation than an HTO.

The classically used LDTA is unreliable in terms of evaluating a deformity if the Cora is in any place other than the distal tibia. The M‐LDTA, on the other hand, is independent of the level of deformity.

When planning a knee or ankle osteotomy, it is mandatory to consider the lower limb in toto as the deformity correction is not a segment‐specific surgery but has repercussions on the whole lower extremity.

## AUTHOR CONTRIBUTIONS

Alessio Maione conceived the study, participated in the study design, coordinated the study and helped to draft the manuscript. Carlo Minoli participated in the study design, performed the statistical analysis, coordinated and helped to draft the manuscript. Matteo Davide Parmigiani performed data collection and helped to draft the manuscript. Martino Travi performed data collection and helped to draft the manuscript. Filippo Calanna participated in the study design and helped to draft the manuscript. Daniele Marcolli participated in the study design and helped to draft the manuscript. Riccardo Compagnoni participated in the study design and helped to draft the manuscript. Paolo Ferrua participated in the study design and helped to draft the manuscript. Massimo Berruto conceived the study, participated in the study design, coordinated the study and helped to draft the manuscript. Pietro Simone Randelli conceived the study, participated in the study design and coordinated the study. All authors read and approved the final manuscript.

## CONFLICT OF INTEREST STATEMENT

The authors declare no conflicts of interest.

## ETHICS STATEMENT

The study was designed based on the criteria of the Declaration of Helsinki and approved by the local ethical committee.

## Data Availability

The data that support the findings of this study are not openly available due to sensitivity reasons and are available from the corresponding author upon reasonable request.
